# Behavioural disturbances as the clinical presentation of Wernicke Encephalopathy: a case report

**DOI:** 10.1192/j.eurpsy.2022.1201

**Published:** 2022-09-01

**Authors:** F. Mayor Sanabria, A. Bermejo Pastor, M. Fernández Fariña, M.E. Expósito Durán, C. Regueiro Martín-Albo, M. Jiménez Cabañas, C. Ortiz Sánchez-Expósito

**Affiliations:** Hospital Clínico San Carlos, Instituto De Psiquiatría Y Salud Mental, Madrid, Spain

## Abstract

**Introduction:**

Wernicke Encephalopathy (WE) is the best characterized neurological complication of thiamine deficiency. Its clinical presentation can be diverse, including hallucinatory or delusional symptoms, and not necessarily associated with the classical triad of WE. This correlates with higher rates of under diagnosis. We present the case of a 72-year-old man with a history of alcoholism who was admitted to the hospital due to behavioural disturbances and subacute delusional ideas of harm.

**Objectives:**

To review the epidemiology and clinical features of WE, as well as its clinical management.

**Methods:**

Review of the literature on WE clinical presentation and management, focusing on psychopathological symptoms, applying the information to this specific case.

**Results:**

Classical triad of WE is only to be found in 10-17% of patients. The most common clinical presentation is changes in mental state (82%), varying from subtle changes in memory, apathy, subtle disorientation or indifference to more severe presentations such as delirium, stupor or coma. Other frequent symptoms include oculomotor dysfunction and gait ataxia. High dose thiamine supplementation therapy has proven effective in preventing clinical progression and permanent neurological damage.

**Conclusions:**

- WE is the most prevalent complication of thiamine deficiency, being associated to alcoholism in 50% of cases. - Changes in mental state is the most frequent form of clinical presentation, not necessarily associated with the classical triad of WE. - WE is a medical emergency that requires high dose thiamine supplementation therapy to prevent permanent neurological damage.

**Disclosure:**

No significant relationships.

